# Development of a core outcome set for breast cancer-related lymphedema: a Delphi study

**DOI:** 10.1007/s10549-024-07262-5

**Published:** 2024-02-29

**Authors:** David Doubblestein, Linda Koehler, Elizabeth Anderson, Nicole Scheiman, Paula Stewart, Mark Schaverien, Jane Armer

**Affiliations:** 1 Department of Physical Therapy, A.T. Still University, Mesa, AZ USA; 2 Department of Rehabilitation Medicine, University of Minnesota, Minneapolis, MN USA; 3 Sinclair School of Nursing, University of Missouri, Columbia, MO USA; 4 Occupational Therapy Assistant Program, Huntington University, Huntington, IN USA; 5 Parkridge Medical Center – Wound Care/Lymphedema Clinic, Parkridge Medical Center, Chattanooga, TN USA; 6 Department of Plastic Surgery, Division of Surgery, MD Anderson Cancer Center, The University of Texas, Houston, TX USA

**Keywords:** Breast cancer-related lymphedema, Core outcome set, Outcome measures

## Abstract

**Purpose:**

For breast cancer survivors (BCS) living with breast cancer-related lymphedema (BCRL), what outcome domains (OD) should be measured to assess the burden of the disease and efficacy of interventions? A Core Outcome Set (COS) that promotes standardized measurement of outcomes within the constraints of time influenced by work environments is essential for patients and the multidisciplinary professionals that manage and research BCRL.

**Methods:**

Using Delphi methodology, a multidisciplinary group of BCRL experts (physical and occupational therapists, physicians, researchers, physical therapist assistants, nurses, and massage therapist) completed two waves of online surveys. BCRL expert respondents that completed the first survey (*n* = 78) had an average of 26.5 years in practice, whereas, respondents who completed the second survey (*n* = 33) had an average of 24.9 years. ODs were included in the COS when consensus thresholds, ranging from 70% to 80%, were met.

**Results:**

A total of 12 ODs made up the COS. Reaching a minimum consensus of 70%; volume, tissue consistency, pain, patient-reported upper quadrant function, patient-reported health-related quality of life, and upper extremity activity and motor control were recommended at different phases of the BCRL continuum in a time-constrained environment. Joint function, flexibility, strength, sensation, mobility and balance, and fatigue met an 80% consensus to be added when time and resources were not constrained.

**Conclusion:**

The COS developed in this study thoroughly captures the burden of BCRL. Using this COS may reduce selective reporting, inconsistency in clinical use, and variability of reporting across interdisciplinary healthcare fields, which manage or research BCRL.

**Supplementary Information:**

The online version contains supplementary material available at 10.1007/s10549-024-07262-5.

## Background

Over 3.8 million breast cancer survivors (BCS) are living in the United States [1]. This long-term survivorship can bring issues of treatment side-effects such as lymphedema. Breast cancer-related lymphedema (BCRL) is a condition that results from the leakage of protein-rich lymphatic fluid from the lymphatic system into surrounding tissue spaces [2]. In the United States, this condition most commonly develops as a result of breast cancer treatments such as lymphadenectomy and irradiation [3]. While BCS incur a lifetime risk of developing this incurable condition, research has shown between 10 and 50% of BCS develop this incurable condition [4–6]. BCRL may negatively impact physical function, psychosocial health, and health-related quality of life (HRQOL) [7]. These negative impacts on HRQOL may present for at least 10 years with peak upper limb morbidity impacts occurring at 3–5 years post onset [7, 8]. Limitations in household, work, and recreational activities are prevalent and are affected by impairments such as arm heaviness, stiffness, weakness, swelling, dynamic imbalances, decreased sensation, and limited kinesthetic awareness [8–11]. Once BCRL develops, clinical treatment by a Certified Lymphedema Therapist (CLT) and daily self-management is critical to optimizing physical function and overall HRQOL. Research has identified key self-management behaviors such as skin care, simple lymphatic drainage, lymphedema exercises, and wear of compression garments [12, 13].

One topic that has not been well studied are the outcome domains (ODs) that clinicians and researchers use to determine if current self-management and therapeutic interventions are efficacious and whether principal instruments to measure ODs are feasible in clinic and/or research settings. Doubblestein et al. [14] investigated 92 outcome measures (OMs) that CLTs may use with BCS living with BCRL. These OMs parallel 14 ODs corresponding to the World Health Organization’s International Classification of Functioning, Disability and Health (ICF) framework. The authors discovered that 95% of CLTs most often used active range of motion, manual muscle testing, circumference measurements converted to volume, sensation, and tissue consistency OMs when assessing BCRL [14]. However, these measures alone limit a specialist’s comprehensive understanding of the chronic condition and related co-morbidities of BCRL, and limit a clinician’s approach to whole-body assessment and interventions. OMs for patient-reported (PR) fatigue, mobility and balance, and upper extremity activity and motor control were least frequently used as revealed in this study [14]. This is concerning for practicing CLTs and also for pre- and post-professional educators and researchers. Extensive choices of ODs and OMs should be narrowed to guide specialists and researchers alike to gather best outcome evidence. Identifying ODs and correlated OMs with good psychometric properties for examination of BCS living with BCRL, including the associated co-morbidities, benefits the BCRL practitioner and researcher regardless of their professional background.

To support evidence-based practice and bring standardization of language across an interdisciplinary care alliance, it is important to identify and define a core outcome set (COS) and a core set of OMs. A COS is a standardized collection of patient ODs which are essential to be collected in clinical trials on a specific medical condition or within a specific medical field [15, 16]. In healthcare, a COS can assist in the examination of a disorder and related comorbidities, and for the purpose of outcome assessment of interventions [17, 18]. The use of a COS can reduce selective reporting on conditions, inconsistency in clinical assessment, and variability of reporting across interdisciplinary healthcare fields which manage or research BCRL [15, 19]. Furthermore, COSs can lead to improved systematic reviews and meta-analyses by facilitating standardization of OMs [15], homogeneity of articles, and robust effect sizes. The groundwork in developing a COS for BCRL and adjuvant core set of OMs has been laid by the American Physical Therapy—Breast Cancer Evaluation Database to Guide Effectiveness (EDGE) Task Force in identifying ODs that align with the ICF framework and OMs with good psychometrics and clinical utility, as well as the knowledge of what OMs are most often used by CLTs [14, 20–23]. Currently, there is no COS for BCRL for clinical trials or for practice patterns. Development of a COS for BCRL will improve the standardization of ODs and OMs, and facilitate the ability to compare findings for the purposes of intervention analyses and pooling data for meta-analyses. The purpose of this study was to conduct a Delphi study to develop a COS for BCRL.

## Materials and methods

### Design

A Delphi study was developed following the Core Outcome Measures in Effectiveness Trial (COMET) Handbook [15]. A Study Management Group (SMG) [15] was formulated by the principal investigator (DD) to develop the Delphi study, which included a multidisciplinary group of BCRL experts who were CLTs, including Physical Therapists (DD and LK), Registered Nurses (JA and EA), and an Occupational Therapist (NS). To further represent primary stakeholders, a Study Advisory Group (SAG) was formed to provide additional expertise on the study development and data interpretation, which included a physiatrist (PS) and a microsurgeon (MS).

The study included a two-phase online survey format. Both surveys (supplemental information A and B) were constructed through Qualtrics Software, Version January 2023 for internet dissemination by email. The surveys were developed by the SMG, who confirmed face validity through survey review. The survey was piloted to the SAG, who substantiated content validity of the surveys. Content validation through expert judgement provides an informed opinion from qualified experts about how well a survey captures all relevant parts it aims to measure [24]. Content validations are generally conducted during the design of a survey and the primary tasks of the study management and study advisory groups were to clarify and modify the individual survey components to support its construct.

Lack of time and resources are frequently reported as barriers to the use of ODs especially when using OMs that measure activities and participation [25]. The SMG took these barriers into consideration during survey development for the first and second surveys to identify (1) ODs recommended in a time-constrained research or clinic environment, and (2) ODs recommended in a research or clinic environment not constrained by time or resources, respectively. Providing surveys that focused on the highly recommended ODs in constrained versus non-constrained environments enabled a novel approach to reconciling differing views. Fourteen ODs [14] were identified by the SMG and SAG for inclusion in the survey, nine of which correlate with the ICF domain of body structures and functions, and five correlate with the ICF domain of activities and participation. For the first survey (i.e., time-constrained), respondents rated the ODs recommendations as high, medium, and low for each phase on the continuum of care for BCRL including (1) pre-surgical, (2) post-surgical, (3) subclinical, (4) acute, and (5) chronic phases of lymphedema in a time-constrained environment (Definitions; see Table 1). In the second survey, respondent then rated their recommended ODs for each phase on the continuum of care in a non-constrained environment. The ODs of stages of lymphedema only appeared for the pre- and post-surgical phase as staging is diagnostic and typically completed by physicians.

**Table 1:** Definitions of phases and outcome domains

Phases	Description
Pre-surgical	Occurring before or in preparation for breast cancer surgery
Post-surgical	Occurring after breast cancer surgery
Sub-clinical Lymphedema	ISL Stage 0 (Latency), Surveillance of a person at risk of manifesting lymphedema
Acute lymphedema	ISL Stage 1 (spontaneously reversible)
Chronic lymphedema	ISL Stage 2 (spontaneously irreversible) and Stage 3 (elephantiasis)

Outcome domains	Description
Joint function	A joint's ability to move throughout the mechanical aspects of its motion, including its physiological and accessory motions
Flexibility	The ability of soft tissues associated with a joint or series of joints to move through pain free and unrestricted shortening and lengthening
Strength	The capacity of a muscle to produce force or withstand pressure
Volume	The amount of space that organic substances (fluid, protein, fat, bone, muscle, etc.) occupies
Pain	Physical suffering or discomfort caused by illness or injury
Sensation	Perception of an object that comes into contact with the body
Tissue consistency	Tissue/skin resistance against the penetration (pushing) of an instrument or finger. This term is defining pitting edema, fibrosis, and induration
Body composition	Height, weight, BMI, and percentages of fat, bone and muscle in human bodies
Stages of lymphedema	A classification system for the progression or regression of lymphedema
Patient-reported health-related quality of life	A report that a patient fills out that characterizes a patient's awareness of the effects of an illness on their life, including the physical and psycho-social aspects
Patient-reported upper quadrant function	A report that a patient fills out that characterizes a patient's awareness of the effects of an illness on the functional abilities of their upper quadrant
Patient-reported fatigue	A report that a patient fills out that characterizes a patient's awareness of the effects of an illness on their mental and or physical tiredness
Mobility and balance	Balance is the ability to control your body position while standing or moving. Mobility is the ability to stand up and walk in a range of environments
Upper extremity activity	
and motor control	The neuromuscular mechanisms to initiate, negotiate, and grade voluntary movement

### Subjects

Purposive recruitment of a heterogeneous group of qualified content experts who would have a summative understanding of BCRL was vital for this Delphi study. Content expert was defined as a professional who had five or more years managing and/or researching BCRL, which was the minimum inclusion criteria. Snowball sampling was instituted with primary survey disseminations through (1) SMG and study advisory group recommended colleague experts, (2) interrelated conferences, and (3) listserv through the Lymphology Association of North America (LANA). Respondents were excluded if they 1) did not provide consent, 2) practiced outside of the United States or Canada, (3) had less than 5 years of experience with BCRL, or (4) survey completion was less than 67% or included only demographic data.

The study received exempt status from the A.T. Still University—Arizona Institutional Review board. After giving written consent, the participants completed the first online survey, which was available for 30 days. Email addresses were gathered from participants who completed the first survey and subsequently received the second survey 6 weeks later, which was also available for 30 days. The second survey was anonymous to encourage engagement. Reminder emails were sent every 2 weeks to encourage participation. Respondents who completed both surveys received a summary of findings and a brief conclusive demographic survey.

### Data analysis

Data were analyzed using IBM SPSS version 29 (Armonk, New York). Participants demographic and practice characteristics were examined (Table 2) and are presented as counts (*n*), means ± standard deviations, and frequencies (%). The surveys allowed respondents to choose more than one OD that applied for each continuum phase of BCRL. To provide a richer source of data toward understanding the respondent’s choice of ODs for a COS, the multiple response feature of SPSS was used to assess the percent of cases and are presented as counts (*n*) and frequencies (%). A criterion is vital to a Delphi study in order to have a consensus among content expert. However, criteria have typically varied across studies, ranging from 60 to 90%, and choices are seldom justified [15]. The SMG established a priori consensus thresholds for each survey. For the first survey, a consensus threshold of 70% was agreed upon for each BCRL phase continuum, due to the narrowly focused independent variable of a time-constrained clinic or research environment. The second survey consensus threshold was raised to 80% due to the broad scope of the independent variable of an environment not constrained by time or resources.

**Table 2:** Respondent and professional characteristics of respondents

First survey respondents (*n* = 78)	
Profession	*n* (%)
Physical therapist	42 (53.85)
Occupational therapist	20 (25.64)
Physician	9 (11.54)
Researcher	3 (3.85)
Physical therapist assistant	2 (2.56)
Advanced practice nurse	1 (1.28)
Massage therapist	1 (1.28)
CLT trained with 135 CE hours	69 (88.46)
LANA credentialed CLT	62 (79.48)
BCRL workload	
Very light (0–20%)	9 (11.54)
Light (21–40%)	18 (23.07)
Moderate (41–60%)	26 (33.34)
Heavy (61–80%)	19 (24.36)
Very heavy (81–100%)	6 (7.69)
Phases of care BCRL continuum respondent manages/researches	
Pre-surgical	40 (51.28)
Post-surgical	71 (91.02)
Subclinical/surveillance	58 (74.36)
Acute	72 (92.31)
Chronic	76 (97.44)
Primary work setting	
Hospital-based outpatient clinic	51 (65.38)
Hospital-based inpatient service	3 (3.85)
Non-hospital-based outpatient clinic	16 (20.51)
Home care/hospice	4 (5.13)
Academic/research facility	4 (5.13)
	Mean ± SD
Years in professional practice	26.46 ± 9.69
Years as CLT	14.44 ± 8.32
Years managing/researching BCRL	17.24 ± 7.41
Second survey respondents (*n* = 33)	
Profession (*n* = 26)	n (%)
Physical therapist	14 (53.85)
Occupational therapist	7 (26.92)
Physician	2 (7.69)
Researcher	2 (7.69)
Advanced practice nurse	1 (3.85)
CLT trained with 135 CE hours (*n* = 26)	24 (92.31)
LANA credentialed CLT (*n* = 26)	19 (73.08)
	Mean ± SD
Years practicing profession (*n* = 26)	24.92 ± 10.13
Years as CLT (*n* = 23)	14.70 ± 6.83
Years managing/researching BCRL (*n* = 23)	15.52 ± 7.59

BCRL-Breast cancer-related lymphedema, CE-Continuing education, CLT-Certified lymphedema therapist, LANA-Lymphology association of North America, SD-Standard Deviation

## Results

### Participants

Approximately 3190 surveys were disseminated, with 133 surveys initiated. The final sample included 78 surveys for data analysis due to incompleteness (*n* = 52), less than 5 years of experience (*n* = 2), and participant from excluded country (*n* = 1) (supplemental information C). Professions of expert respondents included Physical Therapist (PT) (*n* = 42), Occupational Therapist (OT) (*n* = 20), Physician (*n* = 9), BCRL Researcher (*n* = 3), Physical Therapist Assistant (*n* = 2), Advanced Practice Nurse (*n* = 1), and Massage Therapist (*n* = 1). Respondents had an average of 26.5 ± 9.69 years (range 7.0–51.0) practicing in their profession and an average of 17.2 ± 7.41 years (range 5.0–42.0) either managing and/or researching BCRL. Of those respondents, 88.5% (*n* = 69) were CLTs and 79.5% were certified through LANA. Those who were CLTs had retained this credential for an average of 14.4 ± 8.32 years. A majority of the experts described their workload with BCRL was moderate (41–60% of workload) (*n* = 26) and worked in hospital-based outpatient clinics (*n* = 51). Respondent and practice characteristics are presented in Table 2.

Second surveys were disseminated via submitted emails (*n* = 74) and had a response rate of 54% (*n* = 40) (supplemental information C). Due to incompleteness 7 surveys were removed for a total of 33 surveys available for data analysis which included PT (*n* = 14), OT (*n* = 7), Physician (*n* = 2), BCRL Researcher (*n* = 2), and Advanced Practice Provider (*n* = 1) respondents. Respondents had an average of 15.52 ± 7.59 years either managing and/or researching BCRL. A majority of respondents were CLTs (*n* = 24) and were certified through LANA (*n* = 19). Further characteristics are presented in Table 2.

### Recommended outcome measure—constrained work environment

ODs to be assessed in a time-constrained clinical and/or research environment to investigate the ICF domain of body function and structures were included if they met the minimum consensus threshold of 70%. Volume was highly recommended for all phases on the continuum of BCRL; pre-surgical (*n* = 66, 86.8%), post-surgical (*n* = 72, 96%), subclinical (*n* = 71, 95.9%), acute (*n* = 72, 98.6%), and chronic (*n* = 69, 94.5%). Tissue consistency was highly recommended for the following phases: post-surgical (*n* = 53, 70.7%), subclinical (*n* = 62, 83.8%), acute (*n* = 67, 91.8%), and chronic (*n* = 68, 93.2%). Pain was a highly recommended OD for the post-surgical phase (*n* = 55, 73.3%). Highly recommended ODs for body function and structures that met consensus threshold are expanded in Table 3. Moderately recommended ODs included strength for the subclinical (*n* = 49, 73.1%), acute (*n* = 50, 73.5%), and chronic (*n* = 51, 75%) phases.

**Table 3:** Highly recommended BCRL outcome domains for time-constrained environments that measure the ICF domain of body structures and functions

Phase outcome	*n*	% of responses	% of respondents*	Phase outcome	*n*	% of responses	% of respondents^a^
Pre-surgical (*n* = 76)				Acute (73)			
Joint function	43	13.9	56.6	Joint function	21	7.4	28.8
Flexibility	41	13.2	53.9	Flexibility	21	7.4	28.8
Strength	31	10.0	40.8	Strength	8	2.8	11.0
Volume	66	21.3	86.8*	Volume	72	25.5	98.6*
Pain	32	10.3	42.1	Pain	49	17.4	67.1
Sensation	11	3.5	14.5	Sensation	22	7.8	30.1
Tissue consistency	39	12.6	51.3	Tissue consistency	67	23.8	91.8*
Body composition	24	7.7	31.6	Body composition	22	7.8	30.1
Stages of lymphedema	23	7.4	30.3	Chronic (73)			
Post-surgical (*n* = 75)				Joint function	23	7.9	31.5
Joint function	38	10.3	50.7	Flexibility	25	8.6	34.2
Flexibility	45	12.2	60.0	Strength	13	4.5	17.8
Strength	24	6.5	32.0	Volume	69	23.8	94.5*
Volume	72	19.6	96.0*	Pain	39	13.4	53.4
Pain	55	14.9	73.3*	Sensation	18	6.2	24.7
Sensation	25	6.8	33.3	Tissue consistency	68	23.4	93.2*
Tissue consistency	53	14.4	70.7*	Body composition	35	12.1	47.9
Body composition	12	3.3	16.0				
Stages of lymphedema	44	12.0	58.7				
Subclinical/surveillance (74)							
Joint function	23	7.9	31.1				
Flexibility	20	6.9	27.0				
Strength	13	4.5	17.6				
Volume	71	24.5	95.9*				
Pain	42	14.5	56.8				
Sensation	19	6.6	25.7				
Tissue consistency	62	21.4	83.8*				
Body composition	40	13.8	54.1				

BCRL-Breast cancer-related lymphedema, ICF-International classification of functioning, Disability and Health

^a^Multiple responses for “select all”–the percent of respondents that chose each outcome measure as a highly recommended OM for the respective phase

^*^Met the minimum consensus threshold of 70%

ODs to be assessed in a time-constrained clinical and/or research environment to investigate the ICF domain of activities and participation were included if they met the minimum consensus threshold of 70%. PR upper quadrant function was highly recommended for all phases; pre-surgical (*n* = 58, 81.7%), post-surgical (*n* = 58, 82.9%), subclinical (*n* = 60, 84.5%), acute (*n* = 64, 90.1%), and chronic (*n* = 61, 87.1%). PR HRQOL was recommended for the chronic phase (*n* = 57, 81.4%), while upper extremity activity and motor control was recommended for the post-surgical phase (*n* = 50, 71.4%). Highly recommended time-constrained ODs for activities and participation that met consensus threshold are expanded in Table 4. Moderately recommended ODs included mobility and balance for the post-surgical phase (*n* = 48, 72.7%).

**Table 4:** Highly recommended BCRL outcome domains for time-constrained environments that measure the ICF domain of activities and participation

Phase outcome	*n*	% of responses	% of respondents^a^
Pre-surgical (*n* = 71)			
Patient-reported Health-related quality of life	41	24.0	57.7
Patient-reported upper quadrant function	58	33.9	81.7*
Patient-reported fatigue	12	7.0	16.9
Mobility and balance	15	8.8	21.1
Upper extremity activity and motor control	45	26.3	63.4
Post-surgical (*n* = 70)			
Patient-reported Health-related quality of life	41	22.9	58.6
Patient-reported upper quadrant function	58	32.4	82.9*
Patient-reported fatigue	22	12.3	31.4
Mobility and balance	8	4.5	11.4
Upper extremity activity and motor control	50	27.9	71.4*
Subclinical/Surveillance (*n* = 71)			
Patient-reported Health-related quality of life	45	26.8	63.4
Patient-reported upper quadrant function	60	35.7	84.5*
Patient-reported fatigue	13	7.7	18.3
Mobility and balance	7	4.2	9.9
Upper extremity activity and motor control	43	25.6	60.6
Acute (*n* = 71)			
Patient-reported Health-related quality of life	44	24.0	62.0
Patient-reported upper quadrant function	64	35.0	90.1*
Patient-reported fatigue	16	8.7	22.5
Mobility and balance	11	6.0	15.5
Upper extremity activity and motor control	48	26.2	67.6
Chronic (*n* = 70)			
Patient-reported Health-related quality of life	57	30.0	81.4*
Patient-reported upper quadrant function	61	32.1	87.1*
Patient-reported fatigue	15	7.9	21.4
Mobility and balance	12	6.3	17.1
Upper extremity activity and motor control	45	23.7	64.3

BCRL-Breast cancer-related lymphedema, ICF-International classification of functioning, Disability and Health

^a^Multiple responses for “select all”–the percent of respondents that chose each outcome measure as a highly recommended OM for the respective phase

^*^Met the minimum consensus threshold of 70%

### Recommended outcome measure—non-constrained work environment

**Fig. 1:**
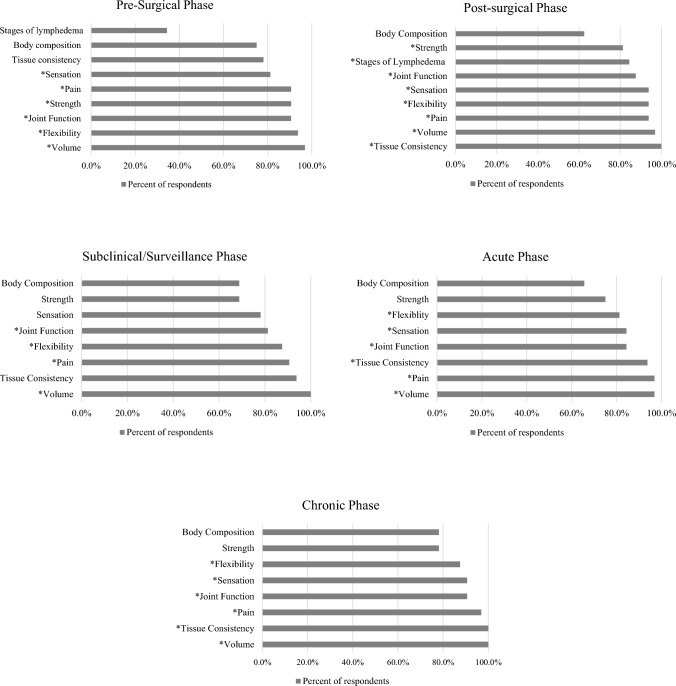
Recommended BCRL outcome domains for non-constrained environments that measure the ICF domain of body structures and functions. BCRL Breast Cancer-Related Lymphedema, ICF International Classification of Disability, Functioning and Health. * Met the minimum consensus threshold of 80%

ODs to be assessed in a clinical and/or research environment not constrained by time or resources to investigate the ICF domain of body function and structures were included if they met the minimum consensus threshold of 80%. Comparable to the time-constrained results, ≥ 93% of respondents highly recommended volume for all phases and tissue consistency was highly recommended for all phases except the pre-surgical phase. Joint function was recommended to be measured for pre-surgical (*n* = 29, 90.6%), post-surgical (*n* = 28, 87.5%), subclinical (*n* = 26, 81.3%), acute (*n* = 27, 84.4%), and chronic (*n* = 29, 90.6%) phases. Flexibility was recommended for pre-surgical (*n* = 30, 93.8%), post-surgical (*n* = 30, 93.8%), subclinical (*n* = 28, 87.5%), acute (*n* = 26, 81.3%), and chronic (*n* = 28, 87.5%) phases. Pain was recommended to be measured for pre-surgical (*n* = 29, 90.6%), post-surgical (*n* = 30, 93.8%), subclinical (*n* = 29, 90.6%), acute (*n* = 31, 96.9%), and chronic (*n* = 31, 96.9%) phases. Sensation did not meet the consensus threshold for the subclinical phase (*n* = 25, 78.1%), and strength did not meet the threshold for subclinical (*n* = 22, 68.8%), acute (*n* = 24, 75%), and chronic (*n* = 25, 78.1%) phases. Recommended ODs to use in a non-constrained work environment for body function and structures that met an 80% consensus threshold are expanded in Fig. 1.

**Fig. 2:**
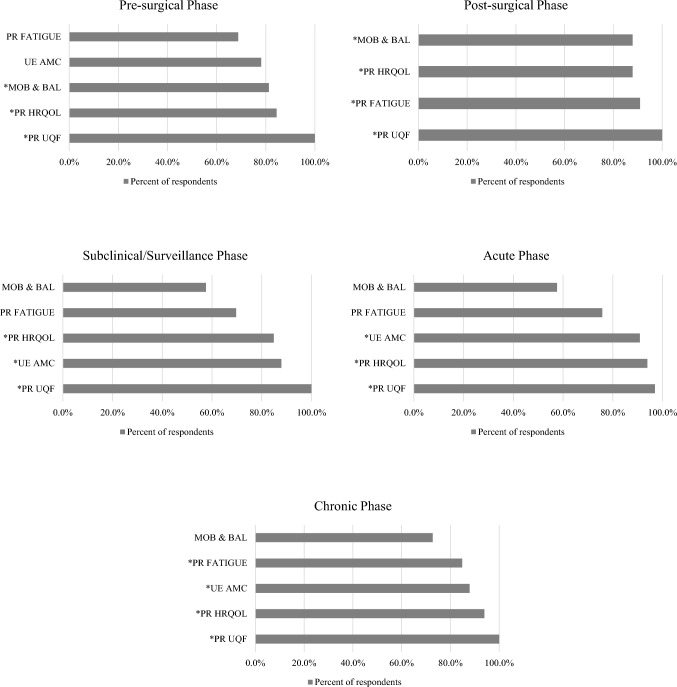
Recommended BCRL outcome domains for non-constrained environments that measure the ICF domain of activities and participation. BCRL Breast Cancer-Related Lymphedema, ICF International Classification of Disability, Functioning and Health, PR Patient-Reported, UE Upper Extremity, AMC Activity and Motor Control, MOB & BAL Mobility and Balance, HRQOL Health-Related Quality of Life, UQF Upper Quadrant Function. * Met the minimum consensus threshold of 80%

ODs to be assessed in a clinical and/or research environment not constrained by time or resources to investigate the ICF domain of activities and participation were included if they met the minimum consensus threshold of 80%. PR HRQOL was recommended to be measured for pre-surgical (*n* = 27, 84.4%), post-surgical (*n* = 29, 87.9%), subclinical (*n* = 28, 84.8%), acute (*n* = 31, 93.9%), and chronic (*n* = 31, 93.9%) phases. PR upper quadrant function was recommended to be measured for pre-surgical (*n* = 32, 100%), post-surgical (*n* = 33, 100%), subclinical (*n* = 33,100%), acute (*n* = 32, 97.0%), and chronic (*n* = 33, 100%) phases. Mobility and balance was recommended to be measured in the pre-surgical (*n* = 26, 81.3%) and post-surgical (*n* = 29, 87.9%) phases. Upper extremity activity and motor control was recommended to be assessed in the post-surgical (*n* = 97.0%), subclinical (*n* = 29, 87.9%), acute (*n* = 30, 90.0%), and chronic phases (*n* = 29, 87.9%). Assessing fatigue was recommended for the post-surgical (*n* = 30, 90.9%) and chronic phases (*n* = 28, 84.8%). Recommended ODs to use in a non-constrained work environment for activities and participation that met an 80% consensus threshold are expanded in Fig. 2.

## Discussion

**Fig. 3:**
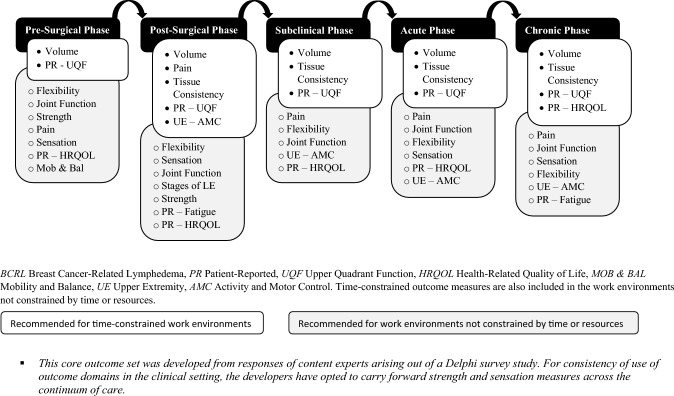
Recommended outcome domains to assess for each phase of the BCRL continuum. BCRL Breast Cancer-Related Lymphedema, PR Patient-Reported, UQF Upper Quadrant Function, HRQOL Health-Related Quality of Life, MOB & BAL Mobility and Balance, UE Upper Extremity, AMC Activity and Motor Control. Time-constrained outcome measures are also included in the work environments not constrained by time or resources

This study sought to establish the minimum COS for guidance and the betterment of BCRL assessments from the perspective of time constraints affecting multidisciplinary clinicians and researchers alike. Gathering measures for multiple ODs that address all comorbidities may be burdensome and unrealistic. Here we present what content experts recommend as a minimum COS for each phase of the BCRL continuum, allowing flexibility for adding further OMs tailored to the clinician’s findings and expertise, as well as the client’s preferences and perspectives. This study proceeded with the awareness that not every profession that manages BCRL has the competence to measure all ODs. However, this fact does not negate the importance of a COS but rather encourages professionals to tap into the strength of a multidisciplinary approach to BCRL care and research. The consensus thresholds were set high to ensure that the COSs were concise for each phase on the BCRL continuum. Twelve ODs were included in the COS. A summary of the COS can be visualized in Fig. 3.

The core OD that received the greatest frequency of recommendations across all phases was volume. Lee et al. and Borman et al. have demonstrated in their studies that changes in volume do not correlate with changes in quality of life [26, 27]. This is a critical point to understand when reflecting on the purpose of a COS. A well-developed minimum core set of outcome domains should equivocally guide the clinician and researcher to abandon singular outcomes to describe the burden of BCRL and embrace ODs that capture important BCRL comorbidities. Tissue consistency was also present across the phases of BCRL except for pre-surgical. These ODs are vital for practice and clinical trials as they assist the professional in staging lymphedema according to the ISL guidelines and the clinical practice guideline published by the Oncology Section of the American Physical Therapy Association [3, 23]. Staging may not be incumbent upon every professional working with BCRL, however, the recommendation to measure these outcomes facilitate proper staging—should this be required.

Assessment of pain was recommended for all phases of the continuum and sensation was recommended for all phases except for the subclinical phase, but did score high at 78%. The multidimensional nature of pain should be considered when interpreting these results. For example, measuring pain in the post-surgical phase may be associated with the concern for acute post-surgical pain and not BCRL since the risk of BCRL is low during this phase. Measuring pain during other phases may be associated with differentiation between BCRL and other causes of pain [28], considering BCRL tends to be described as achy and heavy, but not necessarily ‘painful’[29]. It is evident that BCS with BCRL experience sensory changes, including reduced light touch, static and moving two-point discrimination, pressure pain threshold, and tactile localization sensations, which can improve with complete decongestive therapy [30]. For consistency, the study management and advisory groups advise to consider measuring pain and sensation for all phases.

Strength was not consistently recommended. Strength was recommended to be measured during the post-surgical phase. Perhaps this lack of recommendation correlates with the gap in literature regarding absolute values of weakness of the upper quadrant in BCS with BCRL. However, weakness is self-reported among BCS with BCRL and has also been measured via grip dynamometer [7, 31]. Furthermore, studies are available evidencing the increase in strength with exercise interventions without deleterious effects on BCRL [32]. Despite not meeting the 80% consensus threshold, strength scored high among BCRL experts in the subclinical/surveillance (68%), acute (75%), and chronic (78%) phases. Considering the gap in literature, and strength benefits from therapeutic exercise, the study management and advisory groups encourage measuring strength for all phases on the continuum. 

## Strengths and limitations

This is the first COS for BCRL that extends beyond volume and tissue consistency. The methodology for the development of the survey was a strength in that we used both a study management and advisory group made up of various healthcare disciplines to ensure that the surveys captured the proper content. Respondents were well informed of the purpose of the study and descriptions of all survey content was available via an imbedded hover feature or link to a webpage.

There are limitations that need to be considered when implementing these COSs. First, the established 14 ODs corresponding to the ICF framework were gathered from previous studies and were agreed upon by the SMG and SAG, rather than through a Delphi method. Second, neither the SMG nor SAG included a patient which may have altered the choice of ODs and survey. Third, the retention of experts from the first survey to the second was modest. Due to the steep attrition for the second survey, the SMG decided not to proceed with a third survey and therefore, was unable to resolve the discrepancy on strength and sensation, resulting in the provisional recommendations to measure strength and sensation across the continuum. The study was also restricted to the United States and Canada; therefore, this COS is not intended to be an international guideline. Recommended outcome measures for use with this COS have been identified elsewhere [Accepted]. Finally, while the respondent pool was small given the scope of the study, it comprised a multidisciplinary group of highly experienced practitioners and researchers in BCRL. This COS is considered a living document, where these current recommendations will evolve as the field of lymphedema advances and as OMs develop and become more available.

## Conclusion

Based on this Delphi study, a minimum COS is recommended for BCRL across the continuum of the disease with consideration of the time constraint barrier. Twelve ODs that comprise the COS include volume, tissue consistency, pain, joint function, flexibility, sensation, strength, PR upper quadrant function, PR HRQOL, PR Fatigue, upper extremity activity and motor control, and mobility and balance. This BCRL COS should be used in future clinical trials and is recommended for practicing clinicians to implement in their management of BCRL. To make this COS actionable toward evidence-based practice, steps will need to be initiated toward dissemination and implementation. Obtaining patient input about factors related to this COS is feasible through implementation science. Future research should focus on establishing an international COS for BCRL.

### Supplementary Information

Below is the link to the electronic supplementary material.

Supplementary file1 (DOCX 68 kb)

Supplementary file2 (DOCX 26 kb)

## Data Availability

Not applicable.
