# Supplementary material for: Development of a core outcome set for breast cancer-related lymphedema: a Delphi study
Source: Breast Cancer Res Treat. 2024 Feb 29;205(2):359–70. doi: 10.1007/s10549-024-07262-5 (PMC11101528; doi:10.1007/s10549-024-07262-5)
Supplement: Supplementary file 1 — Supplementary file1 (DOCX 68 kb) [file 10549_2024_7262_MOESM1_ESM.docx]

**Supplemental Information A**

COS on BCRL First Survey

Start of Block: Default Question Block

Q1 **Consent to Participate in a Research Study - Online Survey** Due to your experience and interest in lymphedema, you are invited to take part in this Delphi study. A Delphi study answers a research question by achieving a consensus among experts in the field. The aim of this Delphi study is to develop a Core Outcome Set for breast cancer-related lymphedema. A Core Outcome Set is an agreed standardized set of outcomes recommended to be measured and reported for a specific health condition. The goal is to build this expert consensus through a series of 2 surveys. Each survey will take approximately 15 minutes to complete. This current survey is the first of the 2 surveys. In the event that a consensus is not reached, a third survey will be sent to resolve any discrepancies. Please consider the completion time and survey continuum commitment in your consent to participate. The researchers were careful in using language in the questions when developing the survey to facilitate the ease of understanding and responding.  Keywords have a hover-over feature that will reveal their definitions.  While you may not receive any direct benefit for your participation in this survey, we hope that this study will contribute to the improvement in the evidenced-based practice of breast cancer-related lymphedema evaluations, assessments, and choice of interventions. The survey software has been set so that your email address is retained alongside your survey responses. This feature enables us to send the subsequent survey of this research study and caches survey responses to the participant. Once data has been gathered, the email addresses will be codified for anonymity.
 Participation in this study is completely voluntary. Should you decide to participate now, you may change your mind and stop at any time by simply exiting the survey. We expect to publish the results of this study but will not include any information that would identify you.     **Additional background information about the study, outcome measures, and outcome measure instruments can be found** HERE**.

 If you have questions about this survey, you can contact**
 **Principal Investigator:**  David Doubblestein, PT, PhD
 Email: daviddoubblestein@atsu.edu 
 Phone: 231-629-7109
   **Co-Investigator:** Jane Armer, RN, PhD
 Email: armerj@missouri.edu 

 **As part of their review, A.T. Still University Institutional Review Board has determined that this study is no more than minimal risk and exempt from ongoing IRB oversight.**   **Informed Consent:**
 By clicking on "Yes, I agree to participate," you are consenting to participate in this survey and subsequent surveys of this research study.  

 If you do not wish to participate, select "No, I do not wish to participate" to exit.

Yes, I agree to participate (1)

No, I do not wish to participate (2)

Skip To: End of Survey If Consent to Participate in a Research Study - Online Survey Due to your experience and interest in... = No, I do not wish to participate

Q2 Have you worked with clients with breast cancer-related lymphedema? This can be either in a clinical or research setting.

Yes (1)

No (2)

Skip To: End of Survey If Have you worked with clients with breast cancer-related lymphedema? This can be either in a clini... = No

Q3 **The survey is divided into three sections.  This first section of the survey focuses on demographic and professional information. We are interested in the opinions of experts in the field. There are 21 questions. Please take your time in responding to this survey and thoughtfully consider the questions and answers. You may leave the survey and come back to restart where you left off. For term definitions, please hover your mouse/pointer over the key words of interest.  For best navigation through the survey, we recommend that you use a computer, laptop, or tablet and avoid using a cellular device.**

Q4 What is your profession?

Massage Therapist (3)

Occupational Therapist (5)

Occupational Therapy Assistant (6)

Physical Therapist (7)

Physical Therapist Assistant (8)

Physician (9)

Physician Assistant (10)

Registered Nurse (4)

Other (please describe): (11) __________________________________________________

Q5 How many years have you been practicing your profession?

|  | 0 | 7 | 14 | 21 | 28 | 35 | 42 | 49 | 56 | 63 | 70 |
| --- | --- | --- | --- | --- | --- | --- | --- | --- | --- | --- | --- |

| Years practicing () | 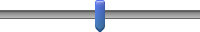 |
| --- | --- |

Q6 What is your highest earned degree?  Select all that apply.

Academic Doctorate (e.g. PhD, D.Sc.) (6)

Clinical Doctorate (e.g. MD, DPT, OTD/DrOT, DNP) (5)

Master of Arts or Science (e.g. MSPT, MOT, MSN) (4)

Bachelor of Arts or Science (e.g. BScPT, BScOT, BScMT, BScN) (3)

Associate of Arts (e.g. PTA, COTA) (2)

Certification/Credentialing (CMT, CBT, CMBT, LMT, RMT) (1)

Q7 Are you a certified lymphedema therapist with 135 hours of training from one educational course?

Yes (1)

No. Please give us details. (2) __________________________________________________

Q8 Are you certified by the Lymphology Association of North America (LANA)?

Yes (1)

No (2)

Q9 How many years have you been certified as a lymphedema therapist?

Please enter years as a whole number (e.g. 5, 10, 20) (1) __________________________________________________

I am not certified (2)

Q10 How many years have you been managing and/or conducting research on breast cancer-related lymphedema?

|  | 0 | 5 | 10 | 15 | 20 | 25 | 30 | 35 | 40 | 45 | 50 |
| --- | --- | --- | --- | --- | --- | --- | --- | --- | --- | --- | --- |

| Click to write Choice 1 () | 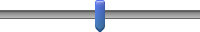 |
| --- | --- |

Q11 When reflecting on your years of experience with breast cancer-related lymphedema, how would you describe the volume of your average workload which was breast cancer-related lymphedema?

Very light (0-20%) (4)

Light (21-40%) (1)

Moderate (41-60%) (2)

Heavy (61-80%) (3)

Very heavy (81-100%) (5)

Q12 Which of the following timelines do you routinely provide assessments and intervention on clients with breast cancer-related lymphedema? Select all that apply.

Pre-surgical breast cancer surgery (1)

Post-surgical breast cancer surgery (2)

Sublinical/Surveillance lymphedema (3)

Acute Lymphedema (4)

Chronic Lymphedema (5)

Q13 Which of the following BEST describes the PRIMARY work setting at which you conduct MOST of your Breast cancer-related lymphedema interactions? (please select only one)

Hospital-based outpatient clinic (1)

Non Hospital-based outpatient clinic (3)

Long-term care facility (6)

Hospital-based inpatient service (2)

Home care/Hospice (4)

Academic / Research facility (5)

Other: please describe. (7) __________________________________________________

Q14 Which of the following best describes the geographical region?

Midwest United States (OH, IN, MI, IL, MO, IA, WI, MN, ND, SD, NE, KS) (1)

Northeast United States (MD, PA, NY, VT, ME, NH, MA, RI, CT, NJ, DE) (2)

Southwest United States (AZ, NM, TX, OK) (3)

Southeast United States (AR, LA, MS, AL, FL, GA, SC, NC, VA, WV, KY, TN, DE) (5)

West United States (CO, UT, WY, MT, ID, WA, OR, NV, CA, AK, HI) (4)

Other: Please describe. (7) __________________________________________________

Q15 Which of the following BEST describes the PRIMARY community setting of your professional practice which you conduct MOST of your Breast cancer-related lymphedema interactions? (please select all that apply)

Urban/Metropolitan (greater than 175,000 inhabitants) (1)

Suburban/Large town (60,000 - 174,999 inhabitants) (2)

Rural (7,500 - 59,999 inhabitants) (3)

Remote rural (less than 7,500 inhabitants) (4)

Q16 This section of the survey focuses on prioritizing outcome measures used to identify Body Structure and Function Impairments along the continuum of breast cancer-related lymphedema disease (pre-surgical, post-surgical, subclinical/surveillance, acute, and chronic). These outcome measures have been established by the APTA Academy of Oncological Physical Therapy and the Dutch Lymphedema Guidelines. For further information, please read the following article: Use of Outcome Measures.  Please answer to your best ability.

Q17 Considering a time-constrained clinical or research environment, please **prioritize** the use of the following outcome measures for the **PRE-SURGICAL** phase on the continuum of care for breast cancer-related lymphedema. *Please move the outcome measure to the level of prioritization (i.e. click and drag to box)*. Click for descriptions.

| High | Medium | Low | I am not trained to assess |
| --- | --- | --- | --- |
| ______ Joint Function (1) | ______ Joint Function (1) | ______ Joint Function (1) | ______ Joint Function (1) |
| ______ Flexibility (2) | ______ Flexibility (2) | ______ Flexibility (2) | ______ Flexibility (2) |
| ______ Strength (3) | ______ Strength (3) | ______ Strength (3) | ______ Strength (3) |
| ______ Volume (4) | ______ Volume (4) | ______ Volume (4) | ______ Volume (4) |
| ______ Pain (5) | ______ Pain (5) | ______ Pain (5) | ______ Pain (5) |
| ______ Sensation (6) | ______ Sensation (6) | ______ Sensation (6) | ______ Sensation (6) |
| ______ Tissue consistency (7) | ______ Tissue consistency (7) | ______ Tissue consistency (7) | ______ Tissue consistency (7) |
| ______ Body composition (8) | ______ Body composition (8) | ______ Body composition (8) | ______ Body composition (8) |
| ______ Stages of lymphedema (9) | ______ Stages of lymphedema (9) | ______ Stages of lymphedema (9) | ______ Stages of lymphedema (9) |

18 Considering a time-constrained clinical or research environment, please **prioritize** the use of the following outcome measures for the **POST-SURGICAL** phase on the continuum of care for breast cancer-related lymphedema. *Please move the outcome measure to the level of prioritization (i.e. click and drag to box)*. Click for descriptions.

| High | Medium | Low | I am not trained to assess |
| --- | --- | --- | --- |
| ______ Joint Function (1) | ______ Joint Function (1) | ______ Joint Function (1) | ______ Joint Function (1) |
| ______ Flexibility (2) | ______ Flexibility (2) | ______ Flexibility (2) | ______ Flexibility (2) |
| ______ Strength (3) | ______ Strength (3) | ______ Strength (3) | ______ Strength (3) |
| ______ Volume (4) | ______ Volume (4) | ______ Volume (4) | ______ Volume (4) |
| ______ Pain (5) | ______ Pain (5) | ______ Pain (5) | ______ Pain (5) |
| ______ Sensation (6) | ______ Sensation (6) | ______ Sensation (6) | ______ Sensation (6) |
| ______ Tissue consistency (7) | ______ Tissue consistency (7) | ______ Tissue consistency (7) | ______ Tissue consistency (7) |
| ______ Body composition (8) | ______ Body composition (8) | ______ Body composition (8) | ______ Body composition (8) |
| ______ Stages of lymphedema (9) | ______ Stages of lymphedema (9) | ______ Stages of lymphedema (9) | ______ Stages of lymphedema (9) |

Q19 Considering a time-constrained clinical or research environment, please **prioritize** the use of the following outcome measures for the **SUBCLINICAL LYMPHEDEMA** phase on the continuum of care for breast cancer-related lymphedema. *Please move the outcome measure to the level of prioritization (i.e. click and drag to box)*. Click for descriptions.

| High | Medium | Low | I am not trained to assess |
| --- | --- | --- | --- |
| ______ Joint Function (1) | ______ Joint Function (1) | ______ Joint Function (1) | ______ Joint Function (1) |
| ______ Flexibility (2) | ______ Flexibility (2) | ______ Flexibility (2) | ______ Flexibility (2) |
| ______ Strength (3) | ______ Strength (3) | ______ Strength (3) | ______ Strength (3) |
| ______ Volume (4) | ______ Volume (4) | ______ Volume (4) | ______ Volume (4) |
| ______ Pain (5) | ______ Pain (5) | ______ Pain (5) | ______ Pain (5) |
| ______ Sensation (6) | ______ Sensation (6) | ______ Sensation (6) | ______ Sensation (6) |
| ______ Tissue consistency (7) | ______ Tissue consistency (7) | ______ Tissue consistency (7) | ______ Tissue consistency (7) |
| ______ Body composition (8) | ______ Body composition (8) | ______ Body composition (8) | ______ Body composition (8) |

Q20 Considering a time-constrained clinical or research environment, please **prioritize** the use of the following outcome measures for the **ACUTE LYMPHEDEMA** phase on the continuum of care for breast cancer-related lymphedema. *Please move the outcome measure to the level of prioritization (i.e. click and drag to box)*. Click for descriptions.

| High | Medium | Low | I am not trained to assess |
| --- | --- | --- | --- |
| ______ Joint Function (1) | ______ Joint Function (1) | ______ Joint Function (1) | ______ Joint Function (1) |
| ______ Flexibility (2) | ______ Flexibility (2) | ______ Flexibility (2) | ______ Flexibility (2) |
| ______ Strength (3) | ______ Strength (3) | ______ Strength (3) | ______ Strength (3) |
| ______ Volume (4) | ______ Volume (4) | ______ Volume (4) | ______ Volume (4) |
| ______ Pain (5) | ______ Pain (5) | ______ Pain (5) | ______ Pain (5) |
| ______ Sensation (6) | ______ Sensation (6) | ______ Sensation (6) | ______ Sensation (6) |
| ______ Tissue consistency (7) | ______ Tissue consistency (7) | ______ Tissue consistency (7) | ______ Tissue consistency (7) |
| ______ Body composition (8) | ______ Body composition (8) | ______ Body composition (8) | ______ Body composition (8) |

Q21 Considering a time-constrained clinical or research environment, please **prioritize** the use of the following outcome measures for the **CHRONIC LYMPHEDEMA** phase on the continuum of care for breast cancer-related lymphedema. *Please move the outcome measure to the level of prioritization (i.e. click and drag to box)*. Click for descriptions.

| High | Medium | Low | I am not trained to assess |
| --- | --- | --- | --- |
| ______ Joint Function (1) | ______ Joint Function (1) | ______ Joint Function (1) | ______ Joint Function (1) |
| ______ Flexibility (2) | ______ Flexibility (2) | ______ Flexibility (2) | ______ Flexibility (2) |
| ______ Strength (3) | ______ Strength (3) | ______ Strength (3) | ______ Strength (3) |
| ______ Volume (4) | ______ Volume (4) | ______ Volume (4) | ______ Volume (4) |
| ______ Pain (5) | ______ Pain (5) | ______ Pain (5) | ______ Pain (5) |
| ______ Sensation (6) | ______ Sensation (6) | ______ Sensation (6) | ______ Sensation (6) |
| ______ Tissue consistency (7) | ______ Tissue consistency (7) | ______ Tissue consistency (7) | ______ Tissue consistency (7) |
| ______ Body composition (8) | ______ Body composition (8) | ______ Body composition (8) | ______ Body composition (8) |

Q22 This section of the survey focuses on prioritizing outcome measures used to identifyActivity Limitations and Participation Restrictions along the continuum of breast cancer-related lymphedema disease (pre-surgical, post-surgical, subclinical/surveillance, acute, and chronic). These outcome measures have been established by the APTA Academy of Oncological Physical Therapy and the Dutch Lymphedema Guidelines. For further information, please read the following article: Use of Outcome Measures. Please answer to your best ability.

Q23 Considering a time-constrained clinical or research environment, please **prioritize** the use of the following outcome measures for the **PRE-SURGICAL** phase on the continuum of care for breast cancer-related lymphedema. *Please move the outcome measure to the level of prioritization (i.e. click and drag to box)*. Click for descriptions.

| High | Medium | Low | I am not trained to assess |
| --- | --- | --- | --- |
| ______ Patient-reported Health-related quality of life (1) | ______ Patient-reported Health-related quality of life (1) | ______ Patient-reported Health-related quality of life (1) | ______ Patient-reported Health-related quality of life (1) |
| ______ Patient-reported upper quadrant function (2) | ______ Patient-reported upper quadrant function (2) | ______ Patient-reported upper quadrant function (2) | ______ Patient-reported upper quadrant function (2) |
| ______ Patient-reported fatigue (3) | ______ Patient-reported fatigue (3) | ______ Patient-reported fatigue (3) | ______ Patient-reported fatigue (3) |
| ______ Mobility and balance (4) | ______ Mobility and balance (4) | ______ Mobility and balance (4) | ______ Mobility and balance (4) |
| ______ Upper extremity activity and motor control (5) | ______ Upper extremity activity and motor control (5) | ______ Upper extremity activity and motor control (5) | ______ Upper extremity activity and motor control (5) |

Q24 Considering a time-constrained clinical or research environment, please **prioritize** the use of the following outcome measures for the **POST-SURGICAL** phase on the continuum of care for breast cancer-related lymphedema. *Please move the outcome measure to the level of prioritization (i.e. click and drag to box)*. Click for descriptions.

| High | Medium | Low | I am not trained to assess |
| --- | --- | --- | --- |
| ______ Patient-reported Health-related quality of life (1) | ______ Patient-reported Health-related quality of life (1) | ______ Patient-reported Health-related quality of life (1) | ______ Patient-reported Health-related quality of life (1) |
| ______ Patient-reported upper quadrant function (2) | ______ Patient-reported upper quadrant function (2) | ______ Patient-reported upper quadrant function (2) | ______ Patient-reported upper quadrant function (2) |
| ______ Patient-reported fatigue (3) | ______ Patient-reported fatigue (3) | ______ Patient-reported fatigue (3) | ______ Patient-reported fatigue (3) |
| ______ Mobility and balance (4) | ______ Mobility and balance (4) | ______ Mobility and balance (4) | ______ Mobility and balance (4) |
| ______ Upper extremity activity and motor control (5) | ______ Upper extremity activity and motor control (5) | ______ Upper extremity activity and motor control (5) | ______ Upper extremity activity and motor control (5) |

Q25 Considering a time-constrained clinical or research environment, please **prioritize** the use of the following outcome measures for the **SUBCLINICAL LYMPHEDEMA** phase on the continuum of care for breast cancer-related lymphedema. *Please move the outcome measure to the level of prioritization (i.e. click and drag to box)*. Click for descriptions.

| High | Medium | Low | I am not trained to assess |
| --- | --- | --- | --- |
| ______ Patient-reported Health-related quality of life (1) | ______ Patient-reported Health-related quality of life (1) | ______ Patient-reported Health-related quality of life (1) | ______ Patient-reported Health-related quality of life (1) |
| ______ Patient-reported upper quadrant function (2) | ______ Patient-reported upper quadrant function (2) | ______ Patient-reported upper quadrant function (2) | ______ Patient-reported upper quadrant function (2) |
| ______ Patient-reported fatigue (3) | ______ Patient-reported fatigue (3) | ______ Patient-reported fatigue (3) | ______ Patient-reported fatigue (3) |
| ______ Mobility and balance (4) | ______ Mobility and balance (4) | ______ Mobility and balance (4) | ______ Mobility and balance (4) |
| ______ Upper extremity activity and motor control (5) | ______ Upper extremity activity and motor control (5) | ______ Upper extremity activity and motor control (5) | ______ Upper extremity activity and motor control (5) |

Q26 Considering a time-constrained clinical or research environment, please **prioritize** the use of the following outcome measures for the **ACUTE LYMPHEDEMA** phase on the continuum of care for breast cancer-related lymphedema. *Please move the outcome measure to the level of prioritization (i.e. click and drag to box)*. Click for descriptions.

| High | Medium | Low | I am not trained to assess |
| --- | --- | --- | --- |
| ______ Patient-reported Health-related quality of life (1) | ______ Patient-reported Health-related quality of life (1) | ______ Patient-reported Health-related quality of life (1) | ______ Patient-reported Health-related quality of life (1) |
| ______ Patient-reported upper quadrant function (2) | ______ Patient-reported upper quadrant function (2) | ______ Patient-reported upper quadrant function (2) | ______ Patient-reported upper quadrant function (2) |
| ______ Patient-reported fatigue (3) | ______ Patient-reported fatigue (3) | ______ Patient-reported fatigue (3) | ______ Patient-reported fatigue (3) |
| ______ Mobility and balance (4) | ______ Mobility and balance (4) | ______ Mobility and balance (4) | ______ Mobility and balance (4) |
| ______ Upper extremity activity and motor control (5) | ______ Upper extremity activity and motor control (5) | ______ Upper extremity activity and motor control (5) | ______ Upper extremity activity and motor control (5) |

Q27 Considering a time-constrained clinical or research environment, please **prioritize** the use of the following outcome measures for the **CHRONIC LYMPHEDEMA** phase on the continuum of care for breast cancer-related lymphedema. *Please move the outcome measure to the level of prioritization (i.e. click and drag to box)*. Click for descriptions.

| High | Medium | Low | I am not trained to assess |
| --- | --- | --- | --- |
| ______ Patient-reported Health-related quality of life (1) | ______ Patient-reported Health-related quality of life (1) | ______ Patient-reported Health-related quality of life (1) | ______ Patient-reported Health-related quality of life (1) |
| ______ Patient-reported upper quadrant function (2) | ______ Patient-reported upper quadrant function (2) | ______ Patient-reported upper quadrant function (2) | ______ Patient-reported upper quadrant function (2) |
| ______ Patient-reported fatigue (3) | ______ Patient-reported fatigue (3) | ______ Patient-reported fatigue (3) | ______ Patient-reported fatigue (3) |
| ______ Mobility and balance (4) | ______ Mobility and balance (4) | ______ Mobility and balance (4) | ______ Mobility and balance (4) |
| ______ Upper extremity activity and motor control (5) | ______ Upper extremity activity and motor control (5) | ______ Upper extremity activity and motor control (5) | ______ Upper extremity activity and motor control (5) |

Q28 We invite you to participate in the second survey of this study. In the space provided, please provide us with the same email address from which you received this survey link. You should receive the second survey within 8 weeks.

**Supplemental Information B**

COS on BCRL Second Survey

Q1 **Consent to Participate**   Thank you for participating in the **first survey** of the Delphi study to develop a Core Outcome Set for Breast Cancer-Related Lymphedema. The preliminary results of the first survey can be found HERE.  

 In this **second survey**, we are interested in knowing what outcome measures you would incorporate into your assessment of a patient with breast cancer-related lymphedema, considering a work environment that is **NOT CONSTRAINED** in time or resources. In addition, we are investigating what instruments you would highly recommend using to measure specific outcome measures and their related feasibility in clinical settings and research settings. The instruments that are listed are not exhaustive. They have been purposefully narrowed down and taken from 92 outcome measures that have been previously investigated. The chosen instruments represent the most frequently used (50 - 100%) instruments by Certified Lymphedema Therapists to measure the outcomes. For further information, please read the following article: Use of Outcome Measures. 
   This survey will take approximately 20 minutes to complete. **Keywords have a hover-over feature** that will reveal their definitions. **Additional background information can be found** HERE**.** The survey software has been set so that your email address is retained alongside your survey responses. Once data has been gathered, the email addresses will be codified for anonymity. Participation in this study is completely voluntary. Should you decide to participate now, you may change your mind and stop at any time by simply exiting the survey. We expect to publish the results of this study but will not include any information that would identify you.     **If you have questions about this survey, you can contact**
 **Principal Investigator:**  David Doubblestein, PT, PhD
 Email: daviddoubblestein@atsu.edu 
 Phone: 231-629-7109
 **Co-Investigator:** Jane Armer, RN, PhD
 Email: armerj@missouri.edu 

 **As part of their review, A.T. Still University Institutional Review Board has determined that this study is no more than minimal risk and exempt from ongoing IRB oversight.**   **Informed Consent:**
 By clicking on "Yes, I agree to participate," you are consenting to participate in this survey.

 If you do not wish to participate, select "No, I do not wish to participate" to exit.

- Yes, I agree to participate (1)
- No, I do not wish to participate (2)

Skip To: End of Survey If Consent to Participate   Thank you for participating in the first survey of the Delphi study to d... = No, I do not wish to participate

Q2 This section of the survey focuses on outcome measures used to identifyBody Structure and Function Impairments along the continuum of breast cancer-related lymphedema (pre-surgical, post-surgical, subclinical/surveillance, acute, and chronic). These outcome measures have been established by the APTA Academy of Oncological Physical Therapy and the Dutch Lymphedema Guidelines. For further information, please read the following article: Use of Outcome Measures.  Please answer to your best ability. For best navigation through the survey, we recommend that you use a computer, laptop, or tablet and avoid using a cellular device.

Q3 Given ample time and resources, which of the following outcome measures would you **include** in your assessment of a patient during the **PRE-SURGICAL** phase on the continuum of care for breast cancer-related lymphedema? *Please move the outcome measure to the appropriate box (i.e. click-drag, and drop in the box). Do not be concerned about ranking the measures*. Click for descriptions.

| Include for this phase | I am not trained to assess | NOT include for this phase |
| --- | --- | --- |
| ______ Joint Function (1) | ______ Joint Function (1) | ______ Joint Function (1) |
| ______ Flexibility (2) | ______ Flexibility (2) | ______ Flexibility (2) |
| ______ Strength (3) | ______ Strength (3) | ______ Strength (3) |
| ______ Volume (4) | ______ Volume (4) | ______ Volume (4) |
| ______ Pain (5) | ______ Pain (5) | ______ Pain (5) |
| ______ Sensation (6) | ______ Sensation (6) | ______ Sensation (6) |
| ______ Tissue consistency (7) | ______ Tissue consistency (7) | ______ Tissue consistency (7) |
| ______ Body composition (8) | ______ Body composition (8) | ______ Body composition (8) |
| ______ Stages of lymphedema (9) | ______ Stages of lymphedema (9) | ______ Stages of lymphedema (9) |

Q4 Given ample time and resources, which of the following outcome measures would you **include** in your assessment of a patient during the **POST-SURGICAL** phase on the continuum of care for breast cancer-related lymphedema? *Please move the outcome measure to the appropriate box (i.e. click-drag, and drop in the box). Do not be concerned about ranking the measures*. Click for descriptions.

| Include for this phase | I am not trained to assess | NOT include for this phase |
| --- | --- | --- |
| ______ Joint Function (1) | ______ Joint Function (1) | ______ Joint Function (1) |
| ______ Flexibility (2) | ______ Flexibility (2) | ______ Flexibility (2) |
| ______ Strength (3) | ______ Strength (3) | ______ Strength (3) |
| ______ Volume (4) | ______ Volume (4) | ______ Volume (4) |
| ______ Pain (5) | ______ Pain (5) | ______ Pain (5) |
| ______ Sensation (6) | ______ Sensation (6) | ______ Sensation (6) |
| ______ Tissue consistency (7) | ______ Tissue consistency (7) | ______ Tissue consistency (7) |
| ______ Body composition (8) | ______ Body composition (8) | ______ Body composition (8) |
| ______ Stages of lymphedema (9) | ______ Stages of lymphedema (9) | ______ Stages of lymphedema (9) |

Q5 Given ample time and resources, which of the following outcome measures would you **include**in your assessment of a patient during the **SUBCLINICAL LYMPHEDEMA** phase on the continuum of care for breast cancer-related lymphedema? *Please move the outcome measure to the appropriate box (i.e. click-drag, and drop in the box). Do not be concerned about ranking the measures*. Click for descriptions.

| Include for this phase | I am not trained to assess | NOT include for this phase |
| --- | --- | --- |
| ______ Joint Function (1) | ______ Joint Function (1) | ______ Joint Function (1) |
| ______ Flexibility (2) | ______ Flexibility (2) | ______ Flexibility (2) |
| ______ Strength (3) | ______ Strength (3) | ______ Strength (3) |
| ______ Volume (4) | ______ Volume (4) | ______ Volume (4) |
| ______ Pain (5) | ______ Pain (5) | ______ Pain (5) |
| ______ Sensation (6) | ______ Sensation (6) | ______ Sensation (6) |
| ______ Tissue consistency (7) | ______ Tissue consistency (7) | ______ Tissue consistency (7) |
| ______ Body composition (8) | ______ Body composition (8) | ______ Body composition (8) |

Q6 Given ample time and resources, which of the following outcome measures would you **include** in your assessment of a patient during the **ACUTE LYMPHEDEMA** phase on the continuum of care for breast cancer-related lymphedema? *Please move the outcome measure to the appropriate box (i.e. click-drag, and drop in the box). Do not be concerned about ranking the measures*. Click for descriptions.

| Include for this phase | I am not trained to assess | NOT include for this phase |
| --- | --- | --- |
| ______ Joint Function (1) | ______ Joint Function (1) | ______ Joint Function (1) |
| ______ Flexibility (2) | ______ Flexibility (2) | ______ Flexibility (2) |
| ______ Strength (3) | ______ Strength (3) | ______ Strength (3) |
| ______ Volume (4) | ______ Volume (4) | ______ Volume (4) |
| ______ Pain (5) | ______ Pain (5) | ______ Pain (5) |
| ______ Sensation (6) | ______ Sensation (6) | ______ Sensation (6) |
| ______ Tissue consistency (7) | ______ Tissue consistency (7) | ______ Tissue consistency (7) |
| ______ Body composition (8) | ______ Body composition (8) | ______ Body composition (8) |

Q7 Given ample time and resources, which of the following outcome measures would you **include** in your assessment of a patient during the **CHRONIC LYMPHEDEMA** phase on the continuum of care for breast cancer-related lymphedema? *Please move the outcome measure to the appropriate box (i.e. click-drag, and drop in the box). Do not be concerned about ranking the measures*. Click for descriptions.

| Include for this phase | I am not trained to assess | NOT include for this phase |
| --- | --- | --- |
| ______ Joint Function (1) | ______ Joint Function (1) | ______ Joint Function (1) |
| ______ Flexibility (2) | ______ Flexibility (2) | ______ Flexibility (2) |
| ______ Strength (3) | ______ Strength (3) | ______ Strength (3) |
| ______ Volume (4) | ______ Volume (4) | ______ Volume (4) |
| ______ Pain (5) | ______ Pain (5) | ______ Pain (5) |
| ______ Sensation (6) | ______ Sensation (6) | ______ Sensation (6) |
| ______ Tissue consistency (7) | ______ Tissue consistency (7) | ______ Tissue consistency (7) |
| ______ Body composition (8) | ______ Body composition (8) | ______ Body composition (8) |

Q8 This section of the survey focuses on outcome measures used to identifyActivity Limitations and Participation Restrictions along the continuum of breast cancer-related lymphedema (pre-surgical, post-surgical, subclinical/surveillance, acute, and chronic). These outcome measures have been established by the APTA Academy of Oncological Physical Therapy and the Dutch Lymphedema Guidelines. For further information, please read the following article: Use of Outcome Measures. Please answer to your best ability.

Q9 Given ample time and resources, which of the following outcome measures would you **include** in your assessment of a patient during the **PRE-SURGICAL** phase on the continuum of care for breast cancer-related lymphedema? *Please move the outcome measure to the appropriate box (i.e. click-drag, and drop in the box). Do not be concerned about ranking the measures*. Click for descriptions.

| Include for this phase | I am not trained to assess | NOT include for this phase |
| --- | --- | --- |
| ______ Patient-reported Health-related quality of life (1) | ______ Patient-reported Health-related quality of life (1) | ______ Patient-reported Health-related quality of life (1) |
| ______ Patient-reported upper quadrant function (2) | ______ Patient-reported upper quadrant function (2) | ______ Patient-reported upper quadrant function (2) |
| ______ Patient-reported fatigue (3) | ______ Patient-reported fatigue (3) | ______ Patient-reported fatigue (3) |
| ______ Mobility and balance (4) | ______ Mobility and balance (4) | ______ Mobility and balance (4) |
| ______ Upper extremity activity and motor control (5) | ______ Upper extremity activity and motor control (5) | ______ Upper extremity activity and motor control (5) |

Q10 Given ample time and resources, which of the following outcome measures would you **include** in your assessment of a patient during the **POST-SURGICAL** phase on the continuum of care for breast cancer-related lymphedema? *Please move the outcome measure to the appropriate box (i.e. click-drag, and drop in the box). Do not be concerned about ranking the measures*. Click for descriptions.

| Include for this phase | I am not trained to assess | NOT include for this phase |
| --- | --- | --- |
| ______ Patient-reported Health-related quality of life (1) | ______ Patient-reported Health-related quality of life (1) | ______ Patient-reported Health-related quality of life (1) |
| ______ Patient-reported upper quadrant function (2) | ______ Patient-reported upper quadrant function (2) | ______ Patient-reported upper quadrant function (2) |
| ______ Patient-reported fatigue (3) | ______ Patient-reported fatigue (3) | ______ Patient-reported fatigue (3) |
| ______ Mobility and balance (4) | ______ Mobility and balance (4) | ______ Mobility and balance (4) |
| ______ Upper extremity activity and motor control (5) | ______ Upper extremity activity and motor control (5) | ______ Upper extremity activity and motor control (5) |

Q11 Given ample time and resources, which of the following outcome measures would you **include**in your assessment of a patient during the **SUBCLINICAL LYMPHEDEMA** phase on the continuum of care for breast cancer-related lymphedema? *Please move the outcome measure to the appropriate box (i.e. click-drag, and drop in the box). Do not be concerned about ranking the measures*. Click for descriptions.

| Include for this phase | I am not trained to assess | NOT include for this phase |
| --- | --- | --- |
| ______ Patient-reported Health-related quality of life (1) | ______ Patient-reported Health-related quality of life (1) | ______ Patient-reported Health-related quality of life (1) |
| ______ Patient-reported upper quadrant function (2) | ______ Patient-reported upper quadrant function (2) | ______ Patient-reported upper quadrant function (2) |
| ______ Patient-reported fatigue (3) | ______ Patient-reported fatigue (3) | ______ Patient-reported fatigue (3) |
| ______ Mobility and balance (4) | ______ Mobility and balance (4) | ______ Mobility and balance (4) |
| ______ Upper extremity activity and motor control (5) | ______ Upper extremity activity and motor control (5) | ______ Upper extremity activity and motor control (5) |

Q12 Given ample time and resources, which of the following outcome measures would you **include** in your assessment of a patient during the **ACUTE LYMPHEDEMA** phase on the continuum of care for breast cancer-related lymphedema? *Please move the outcome measure to the appropriate box (i.e. click-drag, and drop in the box). Do not be concerned about ranking the measures*. Click for descriptions.

| Include for this phase | I am not trained to assess | NOT include for this phase |
| --- | --- | --- |
| ______ Patient-reported Health-related quality of life (1) | ______ Patient-reported Health-related quality of life (1) | ______ Patient-reported Health-related quality of life (1) |
| ______ Patient-reported upper quadrant function (2) | ______ Patient-reported upper quadrant function (2) | ______ Patient-reported upper quadrant function (2) |
| ______ Patient-reported fatigue (3) | ______ Patient-reported fatigue (3) | ______ Patient-reported fatigue (3) |
| ______ Mobility and balance (4) | ______ Mobility and balance (4) | ______ Mobility and balance (4) |
| ______ Upper extremity activity and motor control (5) | ______ Upper extremity activity and motor control (5) | ______ Upper extremity activity and motor control (5) |

Q13 Given ample time and resources, which of the following outcome measures would you **include** in your assessment of a patient during the **CHRONIC LYMPHEDEMA** phase on the continuum of care for breast cancer-related lymphedema? *Please move the outcome measure to the appropriate box (i.e. click-drag, and drop in the box). Do not be concerned about ranking the measures*. Click for descriptions.

| Include for this phase | I am not trained to assess | NOT include for this phase |
| --- | --- | --- |
| ______ Patient-reported Health-related quality of life (1) | ______ Patient-reported Health-related quality of life (1) | ______ Patient-reported Health-related quality of life (1) |
| ______ Patient-reported upper quadrant function (2) | ______ Patient-reported upper quadrant function (2) | ______ Patient-reported upper quadrant function (2) |
| ______ Patient-reported fatigue (3) | ______ Patient-reported fatigue (3) | ______ Patient-reported fatigue (3) |
| ______ Mobility and balance (4) | ______ Mobility and balance (4) | ______ Mobility and balance (4) |
| ______ Upper extremity activity and motor control (5) | ______ Upper extremity activity and motor control (5) | ______ Upper extremity activity and motor control (5) |

Q14 In this section of the second survey we are investigating what instruments you would highly recommend using to measuring the listed outcome measures and their related feasibility (the state of being conveniently used) in clinical settings and research settings. The instruments listed are not exhaustive. They have been purposefully narrowed down and taken from 92 outcome measures that have been previously investigated. The following choices represent the most frequently used (50 - 100%) instruments by Certified Lymphedema Therapists to measure the outcomes. For further information, please read the following article: Use of Outcome Measures. For best navigation through the survey, we recommend that you use a computer, laptop, or tablet and avoid using a cellular device.

Q15 Which of the following outcome measure instruments would you recommend to use to measure **THE PROGRESSION OR REDUCTION OF LYMPHEDEMA** on clients with breast cancer-related lymphedema?  Click here for a description of outcome measures**.**

|  | Highly Recommended (1) | Not as Highly Recommended (2) | Not Recommended (3) | Unfamiliar with instrument (6) |
| --- | --- | --- | --- | --- |
| International Society of Lymphology (ISL) Stages (1) |  |  |  |  |
| Common Terminology Criteria of Adverse Events (CTCAE) (2) |  |  |  |  |
| Upper Extremity Lymphedema Index (UELI) (3) |  |  |  |  |
| Indocyanine Green (ICG) Lymphography (10) |  |  |  |  |
| Magnetic Resonance Lymphangiography (MRL) (11) |  |  |  |  |
| Lymphoscintigraphy (12) |  |  |  |  |

Q16 Please indicate the **FEASIBILITY** of using these outcome measurement tools in the clinical and/or research setting depending on your experience. Click here for a description of outcome measures**.**

|  | Feasible in most outpatient settings (11) | Feasible in most inpatient settings (2) | Feasible in most research settings (6) | No experience (8) |
| --- | --- | --- | --- | --- |
| International Society of Lymphology (ISL) Stages (1) |  |  |  |  |
| Common Terminology Criteria of Adverse Events (CTCAE) (2) |  |  |  |  |
| Upper Extremity Lymphedema Index (UELI) (3) |  |  |  |  |
| Indocyanine Green (ICG) Lymphography (11) |  |  |  |  |
| Magnetic Resonance Lymphangiography (MRL) (12) |  |  |  |  |
| Lymphoscintigraphy (13) |  |  |  |  |

Q17 Which of the following outcome measure instruments would you recommend to use to measure **JOINT FUNCTION** on clients with breast cancer-related lymphedema?  Click here for a description of outcome measures**.**

|  | Highly Recommended (1) | Not as Highly Recommended (2) | Not Recommended (3) | Unfamiliar with instrument (6) |
| --- | --- | --- | --- | --- |
| Dynamic Motion Assessment of Scapula (dichotomous) (1) |  |  |  |  |
| Goniometry - passive range of motion of Upper Extremity (2) |  |  |  |  |
| Goniometry - active range of motion of Upper Extremity (3) |  |  |  |  |

Q18 Please indicate the **FEASIBILITY** of using these outcome measurement tools in the clinical and/or research setting depending on your experience. Click here for a description of outcome measures**.**

|  | Feasible in most outpatient settings (1) | Feasible in most inpatient settings (2) | Feasible in most research settings (7) | No experience (8) |
| --- | --- | --- | --- | --- |
| Dynamic Motion Assessment of Scapula (dichotomous) (1) |  |  |  |  |
| Goniometry - passive range of motion of Upper Extremity (2) |  |  |  |  |
| Goniometry - active range of motion of Upper Extremity (3) |  |  |  |  |

Q19 Which of the following outcome measure instruments would you recommend to measure **FLEXIBILITY** on your clients with breast cancer-related lymphedema? Click here for a description of outcome measures**.**

|  | Highly Recommended (1) | Not as Highly Recommended (2) | Not Recommended (3) | Unfamiliar with instrument (5) |
| --- | --- | --- | --- | --- |
| Pectoralis major length (7) |  |  |  |  |
| Pectoralis minor muscle length (1) |  |  |  |  |
| Stiffness of glenohumeral joint (3) |  |  |  |  |

Q20 Please indicate the **FEASIBILITY** of using these outcome measurement tools in the clinical and/or research setting depending on your experience. Click here for a description of outcome measures**.**

|  | Feasible in most outpatient settings (1) | Feasible in most inpatient settings (2) | Feasible in most research settings (5) | No experience (7) |
| --- | --- | --- | --- | --- |
| Pectoralis major length (7) |  |  |  |  |
| Pectoralis minor muscle length (1) |  |  |  |  |
| Stiffness of glenohumeral joint (3) |  |  |  |  |

Q21 Which of the following outcome measure instruments would you recommend to measure **STRENGTH** on your clients with breast cancer-related lymphedema? Click here for a description of outcome measures**.**

|  | Highly Recommended (1) | Not as Highly Recommended (2) | Not Recommended (3) | Unfamiliar with instrument (5) |
| --- | --- | --- | --- | --- |
| Hand Grip Dynamometry (1) |  |  |  |  |
| Hand Held Dynamometry (2) |  |  |  |  |
| Manual Muscle Testing (MMT) (3) |  |  |  |  |
| Pinch Dynamometry (Tip, Lateral, 3 Jaw Chuck) (5) |  |  |  |  |

Q22 Please indicate the **FEASIBILITY** of using these outcome measurement tools in the clinical and/or research setting depending on your experience. Click here for a description of outcome measures**.**

|  | Feasible in most outpatient settings (1) | Feasible in most inpatient settings (2) | Feasible in most research settings (5) | No experience (7) |
| --- | --- | --- | --- | --- |
| Hand Grip Dynamometry (1) |  |  |  |  |
| Hand Held Dynamometry (2) |  |  |  |  |
| Manual Muscle Testing (MMT) (3) |  |  |  |  |
| Pinch Dynamometry (Tip, Lateral, 3 Jaw Chuck) (5) |  |  |  |  |

Q23 Which of the following outcome measure instruments would you recommend to measure **VOLUME**  on your clients with breast cancer-related lymphedema? Click here for a description of outcome measures**.**

|  | Highly Recommended (1) | Not as Highly Recommended (2) | Not Recommended (3) | Unfamiliar with instrument (5) |
| --- | --- | --- | --- | --- |
| Circumferential Measures - Converted to Volume (2) |  |  |  |  |
| Circumferential Measurements (5) |  |  |  |  |
| Perometry (11) |  |  |  |  |
| Water Displacement (12) |  |  |  |  |

Q24 Please indicate the **FEASIBILITY** of using these outcome measurement tools in the clinical and/or research setting depending on your experience. Click here for a description of outcome measures**.**

|  | Feasible in most outpatient settings (1) | Feasible in most inpatient settings (2) | Feasible in most research settings (5) | No experience (7) |
| --- | --- | --- | --- | --- |
| Circumferential Measures - Converted to Volume (2) |  |  |  |  |
| Circumferential Measurements (5) |  |  |  |  |
| Perometry (11) |  |  |  |  |
| Water Displacement (12) |  |  |  |  |

Q25 Which of the following outcome measure instruments would you recommend to measure **PAIN** on your clients with breast cancer-related lymphedema? Click here for a description of outcome measures**.**

|  | Highly Recommended (1) | Not as Highly Recommended (2) | Not Recommended (3) | Unfamiliar with instrument (5) |
| --- | --- | --- | --- | --- |
| Numeric Pain Rating Scale (5) |  |  |  |  |
| Visual Analog Scale (7) |  |  |  |  |

Q26 Please indicate the **FEASIBILITY** of using these outcome measurement tools in the clinical and/or research setting depending on your experience. Click here for a description of outcome measures**.**

|  | Feasible in most outpatient settings (1) | Feasible in most inpatient settings (2) | Feasible in most research settings (5) | No experience (7) |
| --- | --- | --- | --- | --- |
| Numeric Pain Rating Scale (5) |  |  |  |  |
| Visual Analog Scale (7) |  |  |  |  |

Q27 Which of the following outcome measure instrument would you recommend to measure **SENSATION** on your clients with breast cancer-related lymphedema? Click here for a description of outcome measures**.**

|  | Highly Recommended (1) | Not as Highly Recommended (2) | Not Recommended (3) | Unfamiliar with instrument (5) |
| --- | --- | --- | --- | --- |
| Light Touch (e.g. cotton ball, finger, brush) (1) |  |  |  |  |
| Monofilament (2) |  |  |  |  |
| Sharp-Dull Discrimination (3) |  |  |  |  |
| Two-Point Discrimination (4) |  |  |  |  |

Q28 Please indicate the **FEASIBILITY** of using these outcome measurement tools in the clinical and/or research setting depending on your experience. Click here for a description of outcome measures**.**

|  | Feasible in most outpatient settings (1) | Feasible in most inpatient settings (2) | Feasible in most research settings (5) | No experience (7) |
| --- | --- | --- | --- | --- |
| Light Touch (e.g. cotton ball, finger, brush) (1) |  |  |  |  |
| Monofilament (2) |  |  |  |  |
| Sharp-Dull Discrimination (3) |  |  |  |  |
| Two-Point Discrimination (4) |  |  |  |  |

Q29 Which of the following outcome measure instruments would you recommend to measure **TISSUE CONSISTENCY** on your clients with breast cancer-related lymphedema? Click here for a description of outcome measures**.**

|  | Highly Recommended (1) | Not as Highly Recommended (2) | Not Recommended (3) | Unfamiliar with instrument (5) |
| --- | --- | --- | --- | --- |
| Pitting Edema Test - Palpation (2) |  |  |  |  |
| Tissue Texture - Palpation (normal, soft, spongy, firm) (8) |  |  |  |  |
| Axillary Web Syndrome (10) |  |  |  |  |

Q30 Please indicate the **FEASIBILITY** of using these outcome measurement tools in the clinical and/or research setting depending on your experience. Click here for a description of outcome measures**.**

|  | Feasible in most outpatient settings (1) | Feasible in most inpatient settings (2) | Feasible in most research settings (5) | No experience (7) |
| --- | --- | --- | --- | --- |
| Pitting Edema Test - Palpation (2) |  |  |  |  |
| Tissue Texture - Palpation (normal, soft, spongy, firm) (8) |  |  |  |  |
| Axillary Web Syndrome (10) |  |  |  |  |

Q31 Which of the following outcome measure instruments would you recommend to measure **BODY COMPOSITION** on your clients with breast cancer-related lymphedema? Click here for a description of outcome measures**.**

|  | Highly Recommended (1) | Not as Highly Recommended (2) | Not Recommended (3) | Unfamiliar with instrument (5) |
| --- | --- | --- | --- | --- |
| Body Weight (2) |  |  |  |  |
| Body Mass Index (3) |  |  |  |  |

Q32 Please indicate the **FEASIBILITY** of using these outcome measurement tools in the clinical and/or research setting depending on your experience. Click here for a description of outcome measures**.**

|  | Feasible in most outpatient settings (1) | Feasible in most inpatient settings (2) | Feasible in most research settings (5) | No experience (7) |
| --- | --- | --- | --- | --- |
| Body Weight (2) |  |  |  |  |
| Body Mass Index (3) |  |  |  |  |

Q33 This third section of the survey focuses on outcome measures used to identify Activities and Participation Limitations.  Please answer to your best ability.

Q34 Which of the following **PATIENT-REPORTED** outcome measure instruments would you recommend to measure **HEALTH-RELATED QUALITY OF LIFE** on your clients with breast cancer-related lymphedema? Click here for a description of outcome measures**.**

|  | Highly Recommended (1) | Not as Highly Recommended (2) | Not Recommended (3) | Unfamiliar with instrument (5) |
| --- | --- | --- | --- | --- |
| Lymphedema Functioning, Disability, & Health (Lymph-ICF) (14) |  |  |  |  |
| Functional Assessment of Cancer Therapy - Breast (FACT-B) (4) |  |  |  |  |
| Lymphedema Life Impact Scale (LLIS) (7) |  |  |  |  |
| Lymphedema Quality of Life (LYMQOL) (18) |  |  |  |  |
| Upper Limb Lymphedema 27 (ULL-27) (19) |  |  |  |  |

Q35 Please indicate the **FEASIBILITY** of using these outcome measurement tools in the clinical and/or research setting depending on your experience. Click here for a description of outcome measures**.**

|  | Feasible in most outpatient settings (1) | Feasible in most inpatient settings (2) | Feasible in most research settings (5) | No experience (7) |
| --- | --- | --- | --- | --- |
| Lymphedema Functioning, Disability, & Health (Lymph-ICF) (14) |  |  |  |  |
| Functional Assessment of Cancer Therapy - Breast (FACT-B) (4) |  |  |  |  |
| Lymphedema Life Impact Scale (LLIS) (7) |  |  |  |  |
| Lymphedema Quality of Life (LYMQOL) (18) |  |  |  |  |
| Upper Limb Lymphedema 27 (ULL-27) (19) |  |  |  |  |

Q36 Which of the following **PATIENT-REPORTED** outcome measure instruments would you recommend to measure **UPPER QUADRANT FUNCTION** on your clients with breast cancer-related lymphedema? Click here for a description of outcome measures**.**

|  | Highly Recommended (1) | Not as Highly Recommended (2) | Not Recommended (3) | Unfamiliar with instrument (5) |
| --- | --- | --- | --- | --- |
| Disability of Arm, Shoulder, and Hand Questionnaire (DASH) (2) |  |  |  |  |
| Shoulder Pain and Disability Index (SPADI) (18) |  |  |  |  |
| QuickDASH (10) |  |  |  |  |

Q37 Please indicate the **FEASIBILITY** of using these outcome measurement tools in the clinical and/or research setting depending on your experience. Click here for a description of outcome measures**.**

|  | Feasible in most outpatient settings (1) | Feasible in most inpatient settings (2) | Feasible in most research settings (5) | No experience (7) |
| --- | --- | --- | --- | --- |
| Disability of Arm, Shoulder, and Hand Questionnaire (DASH) (2) |  |  |  |  |
| Shoulder Pain and Disability Index (SPADI) (18) |  |  |  |  |
| QuickDASH (10) |  |  |  |  |

Q38 Which of the following **PATIENT-REPORTED**outcome measure instruments would you recommend to measure **FATIGUE** on your clients with breast cancer-related lymphedema? Click here for a description of outcome measures**.**

|  | Highly Recommended (1) | Not as Highly Recommended (2) | Not Recommended (3) | Unfamiliar with Instrument (5) |
| --- | --- | --- | --- | --- |
| Brief Fatigue Inventory (2) |  |  |  |  |
| Visual Analog Scale - Fatigue (12) |  |  |  |  |

Q39 Please indicate the **FEASIBILITY** of using these outcome measurement tools in the clinical and/or research setting depending on your experience. Click here for a description of outcome measures**.**

|  | Feasible in most outpatient settings (1) | Feasible in most inpatient settings (2) | Feasible in most research settings (5) | No experience (7) |
| --- | --- | --- | --- | --- |
| Brief Fatigue Inventory (2) |  |  |  |  |
| Visual Analog Scale - Fatigue (12) |  |  |  |  |

Q40 Which of the following outcome measure instruments would you recommend to measure **MOBILITY AND BALANCE** on your clients with breast cancer-related lymphedema? Click here for a description of outcome measures**.**

|  | Highly Recommended (1) | Not as Highly Recommended (2) | Not Recommended (3) | Unfamiliar with instrument (5) |
| --- | --- | --- | --- | --- |
| Berg Balance Scale (3) |  |  |  |  |
| Functional Reach Test (11) |  |  |  |  |
| Timed Up and Go (10) |  |  |  |  |
| 5 - Times Sit to Stand (12) |  |  |  |  |
| 6 - Minute Walk Test (13) |  |  |  |  |

Q41 Please indicate the **FEASIBILITY** of using these outcome measurement tools in the clinical and/or research setting depending on your experience. Click here for a description of outcome measures**.**

|  | Feasible in most outpatient settings (1) | Feasible in most inpatient settings (2) | Feasible in most research settings (5) | No experience (7) |
| --- | --- | --- | --- | --- |
| Berg Balance Scale (3) |  |  |  |  |
| Functional Reach Test (11) |  |  |  |  |
| Timed Up and Go (10) |  |  |  |  |
| 5 - Times Sit to Stand (12) |  |  |  |  |
| 6 - Minute Walk Test (13) |  |  |  |  |

Q42 Which of the following outcome measure instruments would you recommend measure **UPPER EXTREMITY ACTIVITY & MOTOR CONTROL** on your clients with breast cancer-related lymphedema? Click here for a description of outcome measures**.**

|  | Highly Recommended (1) | Not as Highly Recommended (2) | Not Recommended (3) | Unfamiliar with instrument (5) |
| --- | --- | --- | --- | --- |
| Purdue Pegboard (4) |  |  |  |  |
| 9 - Hole Peg Test (6) |  |  |  |  |

Q43 Please indicate the **FEASIBILITY** of using these outcome measurement tools in the clinical and/or research setting depending on your experience. Click here for a description of outcome measures**.**

|  | Feasible in most outpatient settings (1) | Feasible in most inpatient settings (2) | Feasible in most research settings (5) | No experience (7) |
| --- | --- | --- | --- | --- |
| Purdue Pegboard (4) |  |  |  |  |
| 9 - Hole Peg Test (6) |  |  |  |  |

Q44 There are tools that measure tissue water content and volume which are not frequently used. However, we need further input from your expertise. Which of the following outcome measure instruments would you recommend to measure **TISSUE WATER CONTENT AND VOLUME** on your clients with breast cancer-related lymphedema?  Click here for a description of outcome measures**.**

|  | Highly Recommended (1) | Not as Highly Recommended (2) | Not Recommended (3) | Unfamiliar with instrument (5) |
| --- | --- | --- | --- | --- |
| Bioelectrical Impedance Analysis (4) |  |  |  |  |
| Tissue Dielectric Constant (12) |  |  |  |  |
| 3D Imaging (15) |  |  |  |  |

Q45 Please indicate the **FEASIBILITY** of using these outcome measurement tools in the clinical and/or research setting depending on your experience. Click here for a description of outcome measures**.**

|  | Feasible in most outpatient settings (1) | Feasible in most inpatient settings (2) | Feasible in most research settings (5) | No experience (7) |
| --- | --- | --- | --- | --- |
| Bioelectrical Impedance Analysis (4) |  |  |  |  |
| Tissue Dielectric Constant (12) |  |  |  |  |
| 3D Imaging (15) |  |  |  |  |

Q46 There are tools not frequently used that measure tissue elasticity/stiffness which gives quantitative values for fibrosis. However, we need further input from your expertise. Which of the following outcome measure instruments would you recommend to measure **TISSUE CONSISTENCY**on your clients with breast cancer-related lymphedema? Click here for a description of outcome measures**.**

|  | Highly Recommended (1) | Not as Highly Recommended (2) | Not Recommended (3) | Unfamiliar with instrument (5) |
| --- | --- | --- | --- | --- |
| Ultrasonography (10) |  |  |  |  |
| Myoton (11) |  |  |  |  |
| SkinFibrometer (13) |  |  |  |  |
| Tonometry (14) |  |  |  |  |

Q47 Please indicate the **FEASIBILITY** of using these outcome measurement tools in the clinical and/or research setting depending on your experience. Click here for a description of outcome measures**.**

|  | Feasible in most outpatient settings (1) | Feasible in most inpatient settings (2) | Feasible in most research settings (5) | No experience (7) |
| --- | --- | --- | --- | --- |
| Ultrasonography (10) |  |  |  |  |
| Myoton (11) |  |  |  |  |
| SkinFibrometer (13) |  |  |  |  |
| Tonometry (14) |  |  |  |  |
