# Supplementary material for: Development of a core outcome set for breast cancer-related lymphedema: a Delphi study
Source: Breast Cancer Res Treat. 2024 Feb 29;205(2):359–70. doi: 10.1007/s10549-024-07262-5 (PMC11101528; doi:10.1007/s10549-024-07262-5)
Supplement: Supplementary file 2 — Supplementary file2 (DOCX 26 kb) [file 10549_2024_7262_MOESM2_ESM.docx]

Supplemental information C. Flow chart of participants.

33 Surveys included in second survey analysis

Excluded with reason

- incompleteness (n = 7)

40 Surveys submitted

78 Surveys included in first survey analysis

74 emails received from first survey.

Sent 74 second survey invitations

Excluded with reasons

- incompleteness (n = 52)
- less than 5 years of experience (n = 2)
- participant from excluded country (n = 1)

3190 Survey invitations

133 Surveys submitted
